# A Rare Case of Seminoma in an Elderly Patient With Suspected Lynch Syndrome

**DOI:** 10.1002/ccr3.72804

**Published:** 2026-06-18

**Authors:** Mostafa Kamandi, Maryam Boozari, Ehsan Soltani, Salman Soltani

**Affiliations:** ^1^ Department of Internal Medicine, Faculty of Medicine Mashhad University of Medical Sciences Mashhad Iran; ^2^ Innovative Medical Research Center, Faculty of Medicine Mashhad Medical Sciences, Islamic Azad University Mashhad Iran; ^3^ Surgical Oncology Research Center Mashhad University of Medical Sciences Mashhad Iran; ^4^ Kidney Transplant Research Center Mashhad University of Medical Sciences Mashhad Iran

**Keywords:** colorectal adenocarcinoma, germ cell tumor, lynch syndrome, seminoma

## Abstract

Lynch syndrome (LS), also known as hereditary nonpolyposis colorectal cancer, is caused by mutations in the mismatch repair genes and confers genetic predisposition to colorectal and other cancers. Germ cell tumors, the majority of which are seminomas, usually arise sporadically and predominantly occur in younger patients; their association with Lynch syndrome rarely occurs. Insight into such associations will provide knowledge about genetic predispositions and their management implications. A 56‐year‐old man was brought in with lower back pain and a testicle mass, and constant pain. Enlarged lymph nodes in the retroperitoneal and paravertebral regions suggested testicular cancer by CT scans. The right radical orchiectomy was performed for him and the tests proved that it was a classic seminoma. He had received four cycles of VIP chemotherapy because the cancer had extended to the lungs. The retroperitoneal lymph node dissection (RPLND) was performed after treatment because some lymph nodes were still enlarged, but no cancer was found in those nodes. Further examinations confirmed that the patient had anemia, and colorectal adenocarcinoma was the result of further investigations. The immunohistochemical analysis revealed that there was a loss of MSH6 protein expression while MLH1, MSH2, and PMS2 were preserved. These findings raise suspicion for Lynch syndrome. This case is interesting because seminoma and colorectal cancer were both identified in a patient with features suggestive of a hereditary cancer syndrome.

## Key Clinical Message

Recognition of genetic predisposition in patients with metachronous malignancies is essential. In cases presenting with germ cell tumors alongside other cancers, evaluation for Lynch syndrome should be considered to guide surveillance and management. However, the lack of molecular confirmation may limit establishing a definitive causal relationship between these malignancies.

## 1 Introduction

Germ cell tumors (GCTs) are a wide variety of neoplasms that develop from primordial germ cells whose occurrence depends on different anatomical locations: gonads or extragonadal. These tumors fall within the category of seminomas or nonseminomas; seminomas are more common in the testes but can also grow outside, for example in the mediastinum and retroperitoneum [1]. The etiology of GCTs entails genetic, environmental, as well as hormonal factors [2]. Seminoma, which is a subtype of GCTs, is usually found within the testes, though it has extragonadal presentations. These tumors hold a uniform cell population and generally are more radio‐sensitive than nonseminoma tumors [3]. While the majority of GCTs are sporadic, there is growing evidence suggesting a potential genetic predisposition in a subset of cases [4].

Lynch syndrome, also known as hereditary nonpolyposis colorectal cancer (HNPCC), is an autosomal dominant disorder characterized by a very high probability of cancer: colorectal and others due to germline mutations in mismatch repair (MMR) genes [5] Although most of the known cancers associated with Lynch syndrome are colorectal and endometrial, there are a few reports of other tumor types including GCTs [6].

In this case report, we present a rare case of a seminoma in a patient with findings suggestive of Lynch syndrome. This case highlights the importance of considering genetic syndromes in the differential diagnosis of GCTs, even in the absence of positive family history [4]. The possible presence of Lynch syndrome in this patient emphasizes the need for genetic counseling and surveillance for associated malignancies in affected individuals and their families [7].

## 2 Case History

### 2.1 Presentation

In January 2023, a 56‐year‐old man entered the Medical Oncology and Hematology Ward of Mashhad University of Medical Sciences with intolerable low back pain along with a soft mass present in the testis. Further history and clinical examination didn't reveal any other abnormal findings besides the above symptoms. The patient's family history was negative for Lynch syndrome‐associated malignancies, with no reports of colorectal, endometrial, or other cancers in first‐degree relatives. Retroperitoneal mass was revealed on the abdominal ultrasound. Work‐up was further carried out with a spiral CT scan of lung, mediastinum, abdomen, and pelvis. Extension of disease to paravertebral and subcarinal lymph nodes with retroperitoneal lymph nodes considerably involve areas. The spiral CT of the lung and mediastinum showed multiple subcarinal area lymphadenopathy, one measuring 30 mm.

**FIGURE 1:**
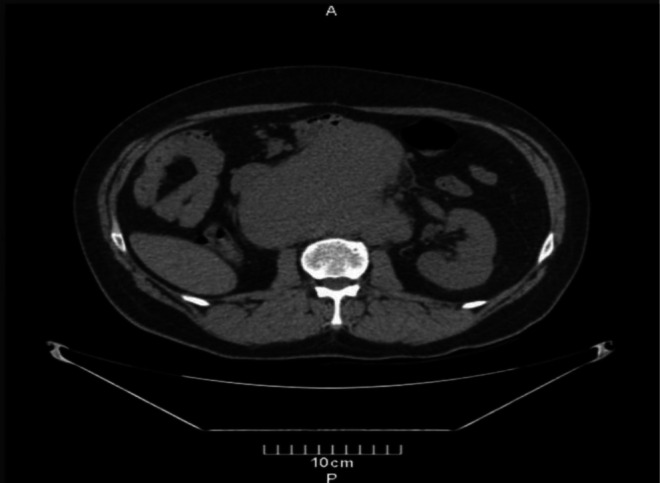
CT scan of abdomen, showing a retroperitoneal mass, suggestive of lymphadenopathy.

Also, the findings in the spiral CT of the abdomen and pelvis revealed massive retroperitoneal adenopathy (Figure 1). In addition, regarding the retrocrural and paravertebral regions, multiple other lymphadenopathy was also seen. In the differential diagnosis of these lesions, relevant lymphoproliferative diseases and metastasis from testis tumor apply. At this time, enough information to confirm the suspicion of testicular neoplasm was given by ultrasound of the scrotum.

### 2.2 Surgical Procedure

He underwent a right radical orchiectomy. The specimen revealed a 6 × 5 × 5 cm tan/white firm circumscribed mass with area of necrosis within the testis. The histologic type was identified as classic seminoma. But because classic seminoma is not common at this age, our clinical suspicion for this patient was lymphoma. To confirm diagnosis, immunohistochemical (IHC) staining for LCA, SALL4 and CD117 was performed.

SALL4 and CD117 were positive, so a definite diagnosis of classic seminoma was reached by immunohistochemistry.

### 2.3 Postoperative Course and Follow‐Up

After orchiectomy, the patient underwent four rounds of VIP chemotherapy instead of standard chemotherapy due to pulmonary involvement. After chemotherapy, the spiral CT scan of the abdomen and pelvis showed a mass measuring 65 × 31 mm. Because the remaining mass was over three centimeters, a *F‐FDG PET/CT scan was performed for restaging. The study showed a hypermetabolic mass in the retroperitoneum at the level of L2‐L3, with a maximum SUV of 5.06. The retrocrural regions showed hypermetabolic lymph nodes on both sides, the maximum SUV being 4.84. There was no abnormality in the liver, spleen, kidneys, adrenals, and thoracic structures. Impressions included residual disease in the retroperitoneal mass. Given that the PET scan results could not assure us of absence of an active tumor, a retroperitoneal lymph node dissection (RPLND) was performed for patient.

RPLND results showed reactive lymph node tissue without tumoral involvement (the results are summarized in Table 1).

**TABLE 1:** Comprehensive analysis of fibroadipose tissue and lymph node findings post‐retroperitoneal lymph node dissection (RPLND).

Specimen	Descriptions	Dimension (cm)	Lymph nodes found	Lymph nodes found	Number of blocks
Aortic and caval lymph node	Fibroadipose tissue	4.5 × 3 × 1.5	2	1–2.3	4
Right gonadal vessel lymph nodes	Fibroadipose tissue	10 × 3 × 1	0	None	5
Retro‐caval lymph nodes	Fibroadipose tissue	6 × 5 × 2.5	6	0.5–2.5	11
Right iliac lymph nodes	Fibroadipose tissue	3.5 × 2 × 1.5 (2 pieces)	2	1–1.5	3
Left anterior aortic lymph nodes	Fibroadipose tissue	3 × 2 × 1	1	1	2
Right retro‐crural lymph nodes	Fibroadipose tissue	5 × 4 × 2.5	2	0.5–0.7	1
Left retro‐crural lymph nodes	Fibroadipose tissue	5 × 3 × 1 (4 pieces)	1	0.6	1

Follow‐up visits every 3 months included CT scans, along with blood tests (CBC, CRT, LDH, ALP) to monitor his condition. On the fourth visit, the patient exhibited pallor associated with increased anemia (Hb: 9.4 g/dL), which worsened with progress. The diagnosis of iron deficiency anemia (IDA) was confirmed by evaluating the iron profile. There seemed to be a necessity for a gastroenterology consultation based on the additional tests done. The gastroenterologist requested a colonoscopy for the patient to justify the patient's iron deficiency anemia.

A colonoscopy revealed narrowing of the lumen and an infiltrative mass of the hepatic flexure. There was a superficial ulcer and invasion into the muscular mucosa as well. The diagnosis was invasive ulcerative adenocarcinoma (well differentiated) as suggested by the hepatic flexure mass biopsy. Immunohistochemical analysis of the colorectal biopsy demonstrated loss of MSH6 protein expression, while MLH1, MSH2, and PMS2 were preserved.

## 3 Discussion and Conclusion

This case highlights the potential association between germ cell tumors and Lynch syndrome, a hereditary condition that increases the risk of various cancers, including colorectal cancer. The patient's presentation with a classic seminoma and subsequent diagnosis of colorectal adenocarcinoma suggests a possible link that warrants further exploration.

Lynch syndrome, also known as hereditary nonpolyposis colorectal cancer (HNPCC), is caused by mutations in mismatch repair (MMR) genes which consequently lead to higher chances of getting several cancers such as colorectal, endometrial, ovarian, and infrequently germ cell. The occurrence of both classic seminoma and subsequent colorectal adenocarcinoma in this patient suggests a possible underlying genetic predisposition [8, 9].

Immunohistochemical analysis showed preserved MLH1, MSH2, and PMS2 expression with isolated loss of MSH6, raising suspicion for Lynch syndrome; however, next‐generation sequencing (NGS) or germline confirmation was not performed, so a clear causal relation cannot be established. Family history was negative for Lynch‐associated and other cancers.

Diagnosis of Lynch syndrome in patients with germ cell tumors has serious clinical ramifications. It may require a holistic approach to patient management, including genetic counseling and regular surveillance for associated malignancies, if relevant to their family history. Finding Lynch syndrome in patients with germ cell tumors will potentially allow for earlier diagnosis and treatment of other cancers and improved overall prognosis [10, 11].

This case suggests that Lynch syndrome should be considered when germ cell tumors are present, especially if there is a simultaneous diagnosis of colorectal cancer. This patient underwent multifaceted management, including surgery, chemotherapy, and regular follow‐up, because of the complexity of her clinical presentation. Further research is still needed to understand the specific relationship between germ cell tumors and Lynch syndrome so that future management approaches can be better tailored for the affected patients [12, 13].

Additionally, the presence of multiple malignancies in a single patient highlights the importance of a thorough and multidisciplinary approach to diagnosis and treatment. The patient's initial presentation with a retroperitoneal mass and subsequent findings of multiple lymphadenopathies required a comprehensive diagnostic work‐up, including imaging and histopathological evaluation. Confirmation of classic seminoma via immunohistochemical staining and subsequent diagnosis of colorectal adenocarcinoma following colonoscopy corroborates the complexity of this patient. This patient was on a regimen of surgery, chemotherapy, and regular discussions regarding follow‐up and suggests an integrated teamwork approach to the management of challenging cases. Advanced imaging mechanisms, such as *F‐FDG PET/CT scans, had important roles in determining the full extent of disease and directing further treatment decisions.

This case, therefore, highlights the importance of genetic syndromes such as Lynch syndrome in the workup of multiple malignancies in patients. Evidence suggests a potential correlation between germ cell tumors and Lynch syndrome, necessitating genetic testing and surveillance to ultimately improve patient outcomes with these syndromes. Future research will need to focus on appropriately defining the connection of these conditions and developing strategies to manage the care of affected patients.

Given the absence of NGS confirmation and the lack of a relevant family history, it is difficult to clearly determine whether there is a true association between the seminoma and Lynch syndrome in this case.

## Author Contributions

**Mostafa Kamandi:** conceptualization, investigation, project administration, writing – review and editing. **Maryam Boozari:** data curation, investigation, writing – original draft, writing – review and editing. **Ehsan Soltani:** writing – review and editing. **Salman Soltani:** writing – review and editing.

## Funding

The authors have nothing to report.

## Consent

The patient's written consent was obtained for the publication of this case report.

## Conflicts of Interest

The authors declare no conflicts of interest.

## Data Availability

The data that support the findings of this study are available from the corresponding author upon reasonable request.
